# The Right to Pay for Hospital Treatment

**Published:** 1910-02-05

**Authors:** 


### Hospital Problems which Press.

### THE RIGHT TO PAY FOR HOSPITAL TREATMENT.

### II.—HOW A VOLUNTARY HOSPITAL MAY INTRODUCE THE PAY SYSTEM.

In order to give practical force to the plan here laid down we beg to formulate for the consideration of every hospital committee the following outline plan whereby all the interests affected can be safeguarded, and the pay system introduced into a voluntary hospital without difficulty or delay. Every voluntary hospital, while admitting all accidents and urgent cases needing immediate attention, should institute a system whereby each applicant would be required to prove that he or she is entitled to receive free treatment. By putting the burden of proof of eligibility to receive free medical relief upon each patient, and the friends, all abuse of every kind must speedily cease. The reforms required in regard to voluntary hospitals and their system of providing for in- and out-patients are that every large hospital shall have connected with the in-patient department, in separate buildings but under the administration of the managers, pay wards for the reception of those patients who are able to pay some portion or the whole cost of their treatment; that, as regards out-patients, the existing out-patient department should be abolished, and that in substitution for it each hospital should have a casualty department and a department for consultation. In the casualty department every applicant should be seen once, and be there disposed of by being handed on to the consultation department, providing the case is found suitable and of sufficient importance. Other cases should be transferred to some provident or Poor Law dispensary, or be referred to a private medical attendant, according to the circumstances of each patient.

#### The Course of Action Suggested.

The voluntary hospitals in each urban community, commencing with London, should nominate a small representative committee, to whom the report of the Royal Commission on the Poor Laws and Relief of Distress (1909) dealing with medical relief should be referred. This committee should have authority to take steps to formulate a scheme whereby:

1. The present overlapping between the voluntary hospitals and the Poor Law in Great Britain in each community will be avoided for the future.
2. A standard will be set up of the medical relief to be provided by each class of existing hospitals, which should secure adequate co-operation between all classes of institutions. In fixing this standard the committee must have clearly before them the facts that the function of the Poor Law is the relief of distress, whilst the object and duty of each voluntary hospital is to apply its resources to the prevention of distress by stepping in to grant free medical relief to the provident and thrifty, who belong entirely to the class of citizens who from no fault of their own may meet with an accident or be overtaken by disease. In this way the present pauperising tendency of medical relief might be entirely eradicated. It is therefore a condition precedent to all reform and reconstruction of voluntary medical institutions that the free relief which they may grant each year shall be strictly confined to those citizens, and those citizens only, who come within the definition which we have just laid down.

#### Principles and Regulations for the Administration of the Pay Wards of Voluntary Hospitals.

To introduce and enforce the pay system adequately it becomes essential that the voluntary hospitals shall remodel the existing plan of granting free medical relief, and reconstitute and rearrange the distribution of the available beds, so as to provide for the admission of citizens above the free patient type who may need hospital care and are prepared to pay for it in varying degree according to their means and circumstances. To enable such a rearrangement to be effectually carried out and to remove the objections which have been raised in various forms to the introduction of the pay system, it is further essential (*a*) that all the principal voluntary hospitals shall adopt the same system; and (*b*) that they shall each be brought into active co-operation with provident and Poor Law dispensaries, and the general body of medical practitioners in the district which each institution serves.

At the commencement of each financial year estimates should be submitted to the committee of (*a*) the probable revenue which will be available during the ensuing twelve months for the treatment and maintenance of patients; (*b*) the number of beds available for patients; (*c*) the average cost of each occupied bed during the ensuing twelve months; and (*d*) the estimated deficiency if all the available beds be treated as free beds. Each committee will thus be placed in a position to decide what proportion of the total number of beds shall be treated as free, and what number shall be assigned to the pay wards. The pay wards then to be grouped on the Swedish system, so as to provide a certain number for the accommodation of each class of pay patients, according to the estimated needs and requirements of each community. We would suggest three classes or grades for pay patients in the voluntary hospitals:

1. Those who can pay a substantial sum for anything and everything a patient may care to have and to pay for, subject to the control of the medical attendant. This class should be provided for in a separate wing, with a separate entrance, so that the medical attendants of these patients who will pay their own fees may be able to see their patients without entering the hospital proper at all.
2. Those patients who will pay a much smaller, but an inclusive rate, which will cover the cost of their treatment during the time they may reside in the hospital; and
3. Patients who desire to pay according to their means towards the cost of their treatment, though they are unable to pay the whole of such cost.

In this way the well-to-do, the self-respecting and the thrifty artisans, in addition to all who are entitled to free medical relief, could be provided for in a great voluntary hospital controlled by one administration, though they would be treated in separate pavilions upon the same site in immediate proximity to each other. The voluntary hospitals may hesitate to face the consideration of reforms as radical as those here suggested, unless they can be guaranteed that the payments by each class of hospital patient in the pay wards will be made in advance. We believe, in practice, regulations might be drawn up to secure this result without any risk of loss; but we have ascertained that it will be easy for the Government or for private enterprise to establish a system of insurance whereby payments for such hospital treatment can be guaranteed through small annual premiums made in the days of health by each of these classes on the basis of the present sickness and accident policies, which are becoming more and more popular with the people of these islands every year. Of course some of the great hospitals may decline to undertake the treatment of patients who can pay a remunerative rate; but we believe that any objection to the reception of such cases would be removed if the municipality were to provide on a business basis the necessary money to defray the cost of the erection of the pay pavilions in connection with a voluntary hospital, whilst every hospital which undertook this work would be able to complete the training of the nursing staff by giving them opportunities of getting adequate experience in private nursing, which at present is practically impossible.

### Safeguards for the Profession.

To meet the just requirements of the medical profession, and to obviate other difficulties, the regulations must include the following safeguards:

1. All pay wards, the patients in which pay the whole cost of their treatment, though under the same administration, must have a separate entrance, and be quite distinct from the free hospital.
2. That the medical staff shall fix the scale of remuneration they are to receive for their services in the pay wards.
3. That no member of any honorary medical staff shall be compelled to undertake the charge of patients in pay wards as part of his duty to the hospital.

Granted that these conditions are enforced, we have reason to believe that some medical men, at any rate, attached to each hospital staff would be willing to see patients in the pay wards at a reduced rate, and that they would consent to a fixed scale of fees.

### The Advantages of this System.

The introduction of a complete rearrangement of the present system of admission of patients to voluntary hospitals upon the lines we have indicated would secure the following advantages:

1. All abuses in connection with the in-patient department of each voluntary hospital would cease.
2. Adequate accommodation would be provided for every class of person who desires hospital treatment for payment in whole or in part.
3. The medical service would be paid for and organised upon a plan which will protect the interests of every member of the medical profession, including members of the honorary medical staff as well as the general practitioner.
4. The wisest economy in administration must prevail.
5. The training of nurses for private work would at length be made efficient.
6. A check would be effectually placed upon the improper growth of medical treatment in hospitals by preventing those who can afford it from obtaining such relief without payment as they do at present.

The institution of pay wards in connection with the voluntary hospitals upon some such basis as is here laid down is desirable, for the reason that by no other means can the present growth in the number of hospital patients be effectually regulated and hospital abuse prevented. Signs are not wanting that unless reforms are speedily forthcoming in the directions we have indicated there is a grave possibility that the rising generation will soon demand free hospital treatment as their birthright, no matter what their means may be, to the great injury of the moral fibre of the nation as a whole. Free medical relief has done infinite mischief to the character of individuals in the past, because it has not exhibited a due regard for the social position of each patient, and has so made against habits of thrift and self-dependence.

---

Dr. H. B. Donkin will deliver the Harveian Oration on St. Luke's Day, and Dr. G. Newton Pitt the Bradshaw Lecture in November, while Dr. A. E. Boycott will give the Milroy Lectures next year.

